# Editorial: Toxic Plant Proteins as Experimental Drugs for Human Pathologies

**DOI:** 10.3389/fphar.2021.689924

**Published:** 2021-04-28

**Authors:** Letizia Polito, Massimo Bortolotti, Rosario Iglesias, Andrea Bolognesi

**Affiliations:** ^1^ Department of Experimental, Diagnostic and Specialty Medicine—DIMES, General Pathology Section, Alma Mater Studiorum—University of Bologna, Bologna, Italy; ^2^ Department of Biochemistry and Molecular Biology and Physiology, Faculty of Sciences, University of Valladolid, Valladolid, Spain

**Keywords:** plant toxins, ribosome-inactivating protein, immunotherapy, balsamin, podophyllotoxin, riproximin, saporin, stenodactylin

**Editorial on the Research Topic** **Toxic Plant Proteins as Experimental Drugs for Human Pathologies**Since the ancient times, plants extracts have been largely used in traditional and folk medicine (Polito et al., 2016a). Some of these medicinal plants are very toxic and their toxicity is often due to the presence of toxic proteins. Among plant toxins, the most known are ribosome-inactivating proteins (RIPs), a family of enzymes widely spread in the plant kingdom, especially in angiosperms, but also present in some fungal and bacterial species (Bolognesi et al., 2016; Polito et al., 2019). RIPs are monomeric (type 1) or dimeric (type 2) proteins depending on the absence or presence of a lectin B-chain linked to the enzymatic A-chain, respectively, being the type 2 RIPs much more toxic for cells. RIP activity was firstly identified as rRNA N-glycosylase. These enzymes were found to remove one specific adenine residue inside the GAGA sequence of the universally conserved sarcin-ricin loop (SRL) of the large rRNA subunit, thus irreversibly damaging ribosomes and causing the arrest of protein synthesis. Afterward, RIPs were also found to be able to deadenylate other substrates, such as genomic DNA, mRNA, tRNA, poly(A) and viral nucleic acids. After linking to appropriate carriers, such as antibodies (immunotoxins), RIPs have been used in experimental therapies to eliminate unwanted cells responsible of pathological conditions with promising results (Polito et al., 2016b). As RIPs have different intracellular substrates and they are able to elicit more than one cell death pathway (Polito et al., 2009; Polito et al., 2016c), they are potential payloads suitable for targeted cancer treatment. Moreover, no drug resistance is reported against RIPs and indeed these molecules were found to be active against cells that had developed multidrug resistance (Dinota et al., 1990). These characteristics make RIPs pharmacologically more attractive than conventional chemotherapy, in which one of the biggest problems is the selection of resistant cells.

The collection of scientific articles composing this Research Topic highlights the progress in the understanding of cell damage mechanisms induced by plant toxins, thus underlying their potential anticancer activity. Moreover, this Research Topic provides an update of the correlations between molecular damages induced by RIPs and the triggering of different cell death pathways.

The type 2 Riproximin, purified from *Ximenia americana*, has demonstrated specific anti-proliferative activity in pancreatic ductal adenocarcinoma cells. Riproximin effects were evaluated in a nude rat model bearing pancreatic cancer cells by human and rat origins, miming both primary and metastatic tumor growth. Gene expression studies showed that Riproximin down-regulated genes involved in cancer progression (Sagini et al.).

Stenodactylin is a highly toxic type 2 RIP purified from the caudex of *Adenia stenodactyla*. The anti-tumor effects of stenodactylin were demonstrated on acute myeloid leukemia cells. Genome-wide gene expression microarray analysis revealed early changes in the expression of genes involved in the regulation of cell death, inflammation and stress response. The early response to stenodactylin treatment was proven to involve inflammatory and apoptotic signaling compatible with the activation of multiple cell death pathways (Mercatelli et al.).

The type 1 RIP balsamin, from *Momordica balsamina*, was combined with some anticancer flavonoids such as naringenin, quercetin and naringin. The treatment with flavonoids plus balsamin reduced HepG2 and MCF-7 cell viability and increased the activation of caspases and induced apoptosis. Balsamin combined with flavonoids also activated endoplasmic reticulum stress–mediated apoptosis in liver and breast cancer cells (Ajji et al.).

A novel suicide gene therapy approach was tested in glioblastoma multiforme cells. The gene coding for saporin was driven intracellularly by a glioma-specific aptamer recognizing the surface antigen nucleolin, efficiently inducing target cell death. Cells that do not expose nucleolin on cell surface, used as control, remained unaffected. Suicide gene therapy was not observed when the inactive saporin mutant DNA was used (di Leandro et al.).

Podophyllotoxin is a plant derivative that has demonstrated an antitumor effect on triple-negative breast cancer cells by inhibiting cell migration and invasion and inducing apoptosis. The Gene Set Enrichment Analysis showed that the expression of some genes often associated with a poor prognosis in breast cancer (i.e., PLK1, CDC20, CDK1) in the cell cycle is inhibited by regulating P53 by podophyllotoxin in triple-negative breast cancer cells (Zhang et al.).

## Conclusion

The plants toxins object of this collection demonstrated antitumor activity on several cancer models of solid tumor and blood cancer. The knowledge of the mechanism(s) of action of these toxins may improve pharmacological strategies to achieve higher specificity and potency in targeting and destroying cancer cells.
